# Statistics and epidemiology of inflammatory bowel disease-associated colorectal neoplasia

**DOI:** 10.1007/s10147-026-02970-y

**Published:** 2026-02-20

**Authors:** Takahide Shinagawa, Satoshi Okada, Hiroshi Shiratori, Yuichi Tachikawa, Yuzo Harada, Yuzo Nagai, Yuichiro Yokoyama, Shigenobu Emoto, Koji Murono, Kazuhito Sasaki, Hiroaki Nozawa, Soichiro Ishihara

**Affiliations:** Department of Surgical Oncology, The University of Tokyo, 7-3-1, Hongo, Bunkyo-Ku, Tokyo 113-8655 Japan

**Keywords:** Inflammatory bowel diseases, Ulcerative colitis, Crohn’s disease, UC-associated neoplasia, CD-associated neoplasia

## Abstract

Inflammatory bowel diseases (IBD), including ulcerative colitis (UC) and Crohn’s disease (CD), are associated with an increased risk of intestinal neoplasia, representing a major long-term complication of chronic inflammation. This review summarizes the recent epidemiological trends and clinicopathological features of IBD-associated colorectal cancer (CRC) and dysplasia in UC and CD. In UC, the cumulative risk of CRC has declined in recent decades, possibly reflecting improvements in medical management and surveillance strategies. However, long disease duration, extensive colitis, concomitant primary sclerosing cholangitis, prior dysplasia, and family history of CRC remain major risk factors. UC-associated neoplasia (UCAN) typically presents as flat lesions with indistinct margins, often accompanied by surrounding dysplasia and frequently exhibits multifocal or infiltrative histological features. The prognosis of UCAN is reportedly poorer than that of sporadic CRC, particularly in advanced stages. In CD, although the overall incidence of neoplasia is lower, the relative risk of colorectal and small intestinal cancer remains significantly higher than in the general population. Geographic variations are notable, with anorectal and fistula-associated carcinomas being most prevalent in East Asia. Risk factors for CD-associated neoplasia (CDAN) include long-standing and early-onset disease, extensive colonic involvement, strictures, and a family history of CRC. Survival outcome of CDAN is worse than that of sporadic CRC, with a higher local recurrence rate. IBD-associated intestinal neoplasia exhibits distinct epidemiological and clinicopathological profiles compared with sporadic CRC. Recent nationwide multicenter studies from Japan provide important insights into UCAN and CDAN, underscoring the importance of region-specific understanding to optimize surveillance and management strategies.

## Introduction

Inflammatory bowel diseases (IBD), such as ulcerative colitis (UC) and Crohn’s disease (CD), are well recognized to increase the risk of colorectal neoplasia [1–3]. UC is a diffuse, nonspecific inflammatory disorder characterized by continuous mucosal inflammation that leads to erosions and ulcers in the colon. In contrast, CD is a transmural inflammatory condition that can affect any part of the gastrointestinal tract, causing complications such as strictures, complex fistulas, and mucosal ulceration. Chronic inflammation of the intestinal mucosa in IBD is associated with accumulation of various genetic and epigenetic alterations that promote neoplastic transformation [4]. Although many aspects of carcinogenesis in patients with IBD are still unclear, its mechanism has been called the “dysplasia-carcinoma sequence’’ [5] and carcinoma, and its precursor, dysplasia, are collectively referred to as IBD-associated intestinal neoplasia. With the increasing global prevalence of IBD, the proportion of patients with IBD-associated intestinal neoplasia, particularly colorectal cancer (CRC) and dysplasia, has become an increasingly important clinical concern [6, 7]. Recently, the Japanese Society for Cancer of the Colon and Rectum (JSCCR) published the 2024 guidelines for the Clinical Practice of Inflammatory Bowel Disease-Associated Intestinal Neoplasia, providing a nationwide consensus on the diagnosis and management of these conditions in Japan [8]. Additionally, a nationwide multicenter database project conducted by the JSCCR elucidated the clinical characteristics and oncological outcomes of IBD-associated intestinal neoplasias, including UC and CD, through a series of primary and secondary analyses [9].

In this review, we provide an overview of current knowledge regarding the statistics and epidemiology of IBD-associated intestinal neoplasia, with a particular focus on colitis-associated CRC and dysplasia in UC and CD.

### UC-associated neoplasia (UCAN)

#### Epidemiology

**Fig. 1:**
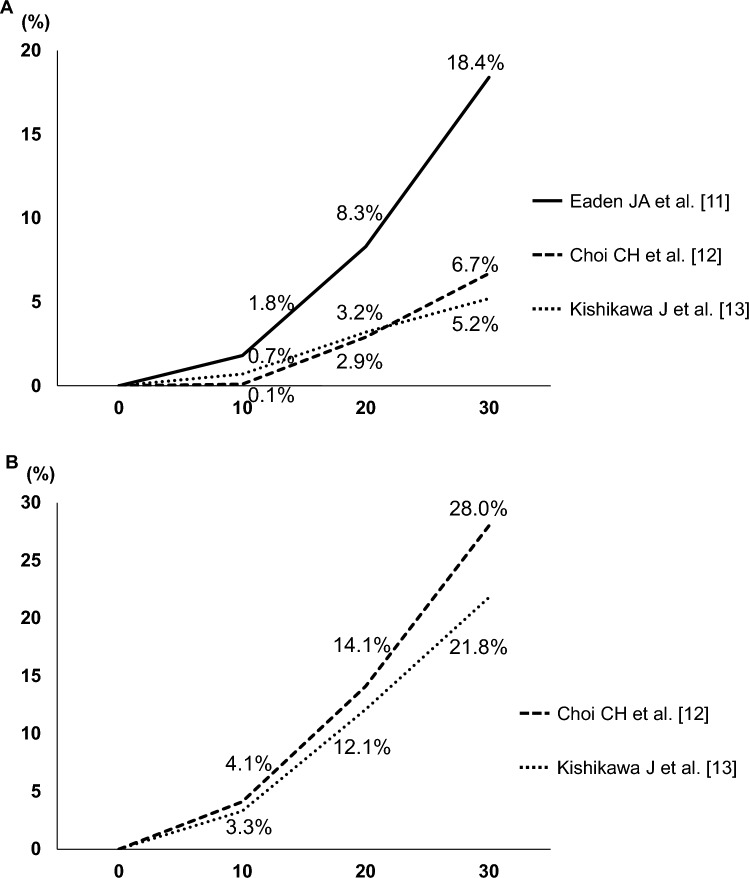
Cumulative incidence of invasive colorectal cancer (**A**) and neoplasia, including low- and high-grade dysplasia (**B**). **A** Eaden et al. reported cumulative incidences of 1.8%, 8.3%, and 18.4% at 10, 20, and 30 years, respectively [11]. In contrast, Choi et al. reported lower rates of 0.1%, 2.9%, and 6.7% at 10, 20, and 30 years, respectively [12], whereas Kishikawa et al. reported rates of 0.7%, 3.2%, and 5.2% at 10, 20, and 30 years, respectively [13]. **B** When dysplasia was included, Choi et al. reported cumulative incidences of 4.1%, 14.1%, 28.0% at 10, 20, and 30 years, respectively [12], whereas Kishikawa et al. reported 3.3%, 12.1%, and 21.8% at 10, 20, and 30 years, respectively [13]

Chronic mucosal inflammation associated with chronic UC is a well-established risk factor for colorectal carcinogenesis. The first case of UCAN was reported by Crohn and Rosenberg in 1925 [10], and subsequent studies have consistently documented this association. The risk of UCAN, including CRC and dysplasia, increases with disease duration. In a meta-analysis by Eaden et al., the cumulative incidence of invasive cancer was 1.8% at 10 years, 8.3% at 20 years, and 18.4% at 30 years [11]. More recent studies suggest lower incidences; In the United Kingdom, Choi et al. reported cumulative incidences of 0.1% at 10 years, 2.9% at 20 years, and 6.7% at 30 years [12]. Similarly, in Japan, Kishikawa et al. reported a cumulative incidence of invasive cancer of 0.7% at 10 years, 3.2% at 20 years, and 5.2% at 30 years, comparable with the findings of Choi et al. (Fig. 1A). The cumulative incidence of dysplasia increased to 3.3%, 12.1%, and 21.8% at 10, 20, and 30 years, respectively, emphasizing the importance of long-term surveillance [13] (Fig. 1B). This trend was supported by a meta-analysis in Asian population, which reported a pooled prevalence of CRC of 0.85% [95% confidence interval (CI), 0.65–1.04] in Asian countries [6]. Another meta-analysis by Jess et al. reported that patients with UC have a 2.4-fold higher risk of developing CRC than the general population [14]. In addition to disease duration, the extent of colonic inflammation is a major determinant of cancer risk. Ekbom et al. reported a relative risk (RR) of CRC of 1.7 for proctitis, 2.8 for left-sided colitis, and 14.8 for extensive colitis compared with the general population, highlighting the need for careful surveillance in patients with widespread disease [1]. Overall, long-term extensive mucosal inflammation is a key risk factor for UCAN development.

### Risk factors

In addition to the disease duration and extent, several other risk factors for UCAN have been identified. These include the severity of inflammation (moderate to severe) (odds ratio, 2.51; 95% CI, 1.75–3.61), coexistence of primary sclerosing cholangitis (PSC) (odds ratio, 4.05; 95% CI, 2.15–7.64), history of dysplasia (odds ratio, 3.64; 95% CI, 1.81–7.32), family history of CRC (odds ratio, 2.42; 95% CI, 1.14–5.16) especially diagnosed at a young age (< 50 years) [15], and presence of strictures (odds ratio, 7.78; 95% CI, 3.74–16.18) [16]. Patients with these risk factors require careful follow-up, including annual surveillance colonoscopies. In particular, patients with concomitant PSC are known to be at a high risk of developing CRC, even at an early stage of the disease, and many guidelines recommend initiating surveillance at the time of PSC diagnosis [17]. A recent secondary analysis of a multicenter database study by the JSCCR project revealed that patients with UCAN and PSC in Japan showed earlier and younger development of UCAN, especially with a higher prevalence in the right-sided colon [18]. Surveillance recommendations for IBD-associated colorectal neoplasia differ slightly among major guidelines. The American Society for Gastrointestinal Endoscopy (ASGE) and the American Gastroenterological Association (AGA) generally recommend initiating surveillance colonoscopy 8–10 years after disease onset in patients with extensive or left-sided colitis, with subsequent intervals stratified by colorectal cancer risk factors. The European Crohn’s and Colitis Organization (ECCO) guidelines adopt a more explicit risk-based approach, recommending annual surveillance for high-risk patients as described above, surveillance every 2 years for intermediate-risk patients, and every 3 years for low-risk patients. Despite regional differences, all guidelines emphasize individualized surveillance strategies based on disease extent, inflammatory burden, and additional risk factors such as PSC or prior dysplasia [17] [19].

### Clinicopathological features

UCAN exhibits several clinicopathological features that distinguish it from sporadic CRC. These lesions frequently present as flat lesions with indistinct margins, in contrast to sporadic CRC, and background mucosal inflammation often makes it difficult to detect lesions and accurately delineate the tumor extent using conventional endoscopy. Therefore, its early detection is challenging. UCAN also tends to be multifocal, and in some cases, exhibits diffuse infiltration into deeper layers despite only minimal epithelial changes on the surface. Furthermore, invisible dysplastic foci may extend around the main tumor, even when the primary lesion is small [20]. The use of high-resolution endoscopic systems and adjunctive chromoendoscopy with indigo carmine has therefore been recommended as these techniques improve lesion detection [19]. In Japan, a multicenter randomized controlled trial compared the detection rates of neoplastic lesions between random and targeted biopsies. The study demonstrated that targeted biopsy achieved a comparable detection rate of neoplastic lesions (11.4% vs. 9.3%) with fewer biopsy samples and a shorter procedure time than random biopsy [21]. Based on these findings, targeted biopsy, —focusing on suspicious lesions identified through detailed inspection using techniques such as chromoendoscopy or magnifying endoscopy—is recommended. However, some macroscopically undetectable lesions can only be identified using random biopsies. Therefore, random biopsy should still be considered depending on the patient’s clinical history and endoscopic findings.

**Fig. 2:**
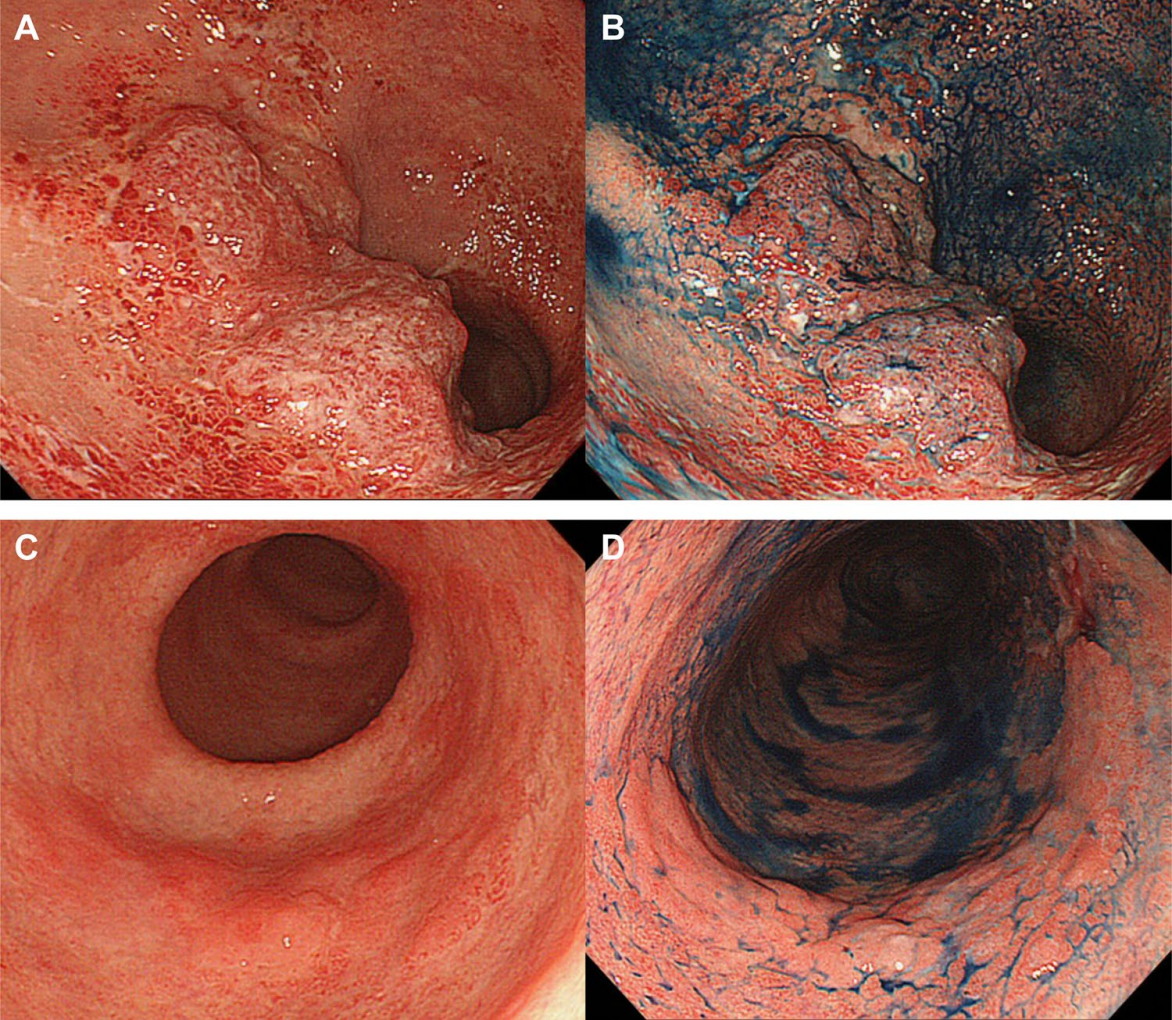
Representative endoscopic findings of UCAN. Upper row: A representative case of type 3 advanced-stage UCAN located in the rectum, observed with **A** white-light endoscopy and **B** indigo carmine chromoendoscopy. Lower row: A representative case of type 0–IIa early-stage UCAN located in the sigmoid colon, observed with **C** white-light endoscopy and **D** indigo carmine chromoendoscopy. Indigo carmine chromoendoscopy is helpful for delineating tumor margins, which are sometimes difficult to identify using white-light endoscopy alone

**Fig. 3:**
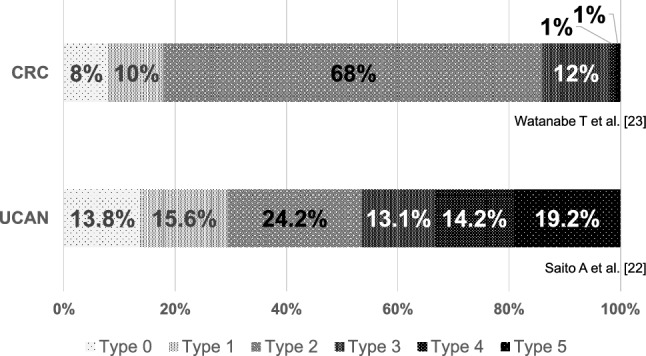
Comparison of macroscopic findings between UCAN and sporadic CRC. In UCAN, type 3, 4, and 5 tumors were more frequent than type 2 tumors (46.5% vs. 24.2%) [22], although type 2 tumors were dominant in sporadic CRC (68%) [23]. UC, ulcerative colitis; UCAN, ulcerative colitis-associated colorectal neoplasia; CRC, colorectal cancer

The macroscopic features of UCAN vary according to the lesion type. Dysplastic lesions sometimes appear as flat, low-elevated, granular, nodular, or irregular flat elevations. In contrast, advanced CRCs in UC are frequently classified as diffusely infiltrating tumors with undermined ulcers (Fig. 2). Therefore, another secondary analysis of the JSCCR database revealed that type 3 (ulcerated with infiltration type), 4 (diffusely infiltrating type), or 5 (unclassified type) tumors occur more frequently than type 2 (ulcerated with clear margin type) tumors in UCAN (46.5% vs. 24.2%) [22] (Table 1), whether type 2 tumors were dominant in sporadic CRC (68.0%) [23] (Fig. 3). Furthermore, among invasive cancers, 13.8% were classified as type 0 preoperatively [22]. Discrimination from sporadic colorectal neoplasia is based on a comprehensive assessment of endoscopic findings including color, margins, and surface structure. Background mucosal changes within the inflamed area of UC, suggesting a history of severe inflammation, supports the diagnosis of UC-associated lesions. Characteristic endoscopic features include irregularly margined flat or granular elevations, villous projections, and localized erythematous areas [19]. In addition to white-light endoscopy, image-enhanced techniques such as narrow-band imaging or blue-laser imaging, as well as chromoendoscopy with indigo carmine or crystal violet, may be useful for refining the diagnosis.

**Table 1:** Summary of characteristics of UCAN and CDAN

	UCAN	CDAN	*P* value
Tumor location [24]			
Ileum	0%	9%	< 0.01
Right-sided colon (C/A/T)	16%	8%	
Left-sided colon (D/S)	31%	7%	
Rectum	51%	28%	
Anal canal/anus	2%	48%	
Macroscopic classification [22]			
Type 0/1/2/3/4/5	14/16/24/13/14/19%	N.A	N.A
Histological type [9]			
wel/mod	73%	43%	< 0.01
por/muc/sig	16%	50%	
Others	11%	7%	
Pathological stage [9]			
0/I/II/III/IV	32/29/17/17/5%	10/17/37/22/14%	< 0.01
Stages in surveillance cases [9]			
0/I	73%	42%	< 0.01
II/III/IV	27%	58%	
Prognosis (5-year OS) [9]			
All stage	87%	59%	< 0.01
Stage 0	97%	100%	0.89
Stage I	95%	90%	0.73
Stage II	89%	76%	0.01
Stage III	68%	18%	< 0.01
Stage IV	13%	0%	0.04

*UCAN* ulcerative colitis-associated colorectal neoplasia, *CDAN* Crohn’s disease-associated neoplasia, *C* cecum, *A* ascending colon, *T* transvers colon, *D* descending colon, *S* sigmoid colon, *wel* well-differentiated adenocarcinoma, *mod* moderately-differentiated adenocarcinoma, *por* poorly differentiated adenocarcinoma, *muc* mucinous adenocarcinoma, *sig* signet-ring cell carcinoma, *OS* overall survival

**Fig. 4:**
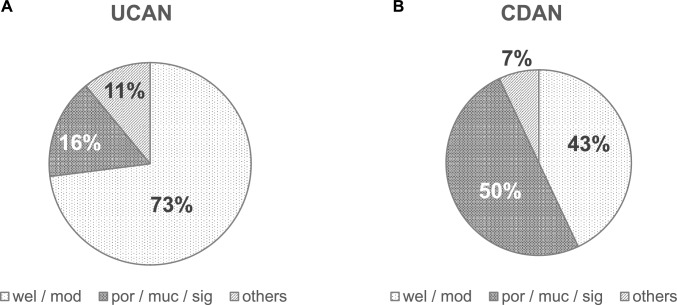
Histological types of UCAN and CDAN. **A** In UCAN, well- or moderately-differentiated adenocarcinomas (wel/mod) accounted for the majority (73%), whereas poorly differentiated adenocarcinoma (por), mucinous adenocarcinoma (muc), and signet-ring cell carcinoma (sig) comprised approximately 16% of cases [9]. **B** In CDAN, por, muc, or sig accounted for about 50% of cases [9]. UCAN, ulcerative colitis-associated colorectal neoplasia; CDAN, Crohn’s disease-associated neoplasia

**Fig. 5:**
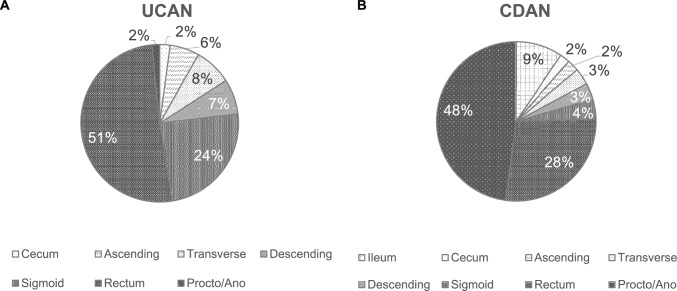
Location of main tumor of UCAN and CDAN. **A** In UCAN, a large majority of tumors were located in the left-sided colon compared with the right-sided colon (84% vs. 16%) [24]. **B** In CDAN, 83% of lesions were located in the left-sided colon, with particularly high involvement of the anal region (48%) [24]. UCAN, ulcerative colitis-associated colorectal neoplasia; CDAN, Crohn’s disease-associated neoplasia

**Fig. 6:**
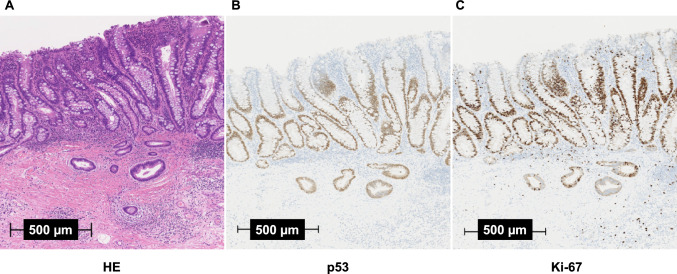
Immunohistochemical examination of UCAN using p53 and Ki-67. **A** Hematoxylin and eosin (HE) staining demonstrates submucosal invasion of UCAN. **B** p53 protein overexpression is predominantly observed in the deep portion of the atypical glands in UCAN at an early phase of carcinogenesis. **C** Ki-67 immunostaining in the same UCAN lesion shows that the cellular proliferation zone is located in the deep to middle layers of the mucosa (bottom-up pattern), whereas in sporadic adenoma, the proliferation zone is typically distributed from the surface to the middle layers of the glands (top-down pattern). UCAN, ulcerative colitis-associated colorectal neoplasia

Histologically, UCAN exhibited a higher frequency of poorly differentiated adenocarcinoma, mucinous adenocarcinoma, and signet-ring cell carcinoma than sporadic CRC (Fig. 4A) (Table 1). These histological differences have recently been reported to become more pronounced with tumor progression, and are more frequently observed in the left-sided colon than in the right-sided colon [24, 25]. It is also noteworthy that a large majority of tumors were located in the left-sided colon, accounting for 84% of cases compared with 16% in the right-sided colon [24] (Fig. 5A) (Table 1). Its carcinogenic pathway differs from that of CRC. In UC, chronic inflammation leads to oxidative stress-induced DNA damage, resulting in the accumulation of genomic and epigenomic alterations including *TP53* mutations. This process gives rise to dysplasia, a recognized precancerous lesion, and subsequently to carcinoma, following the so-called “dysplasia–carcinoma sequence” [26]. Dysplasia has been broadly classified as indefinite dysplasia, low-grade dysplasia, and high-grade dysplasia by Riddell et al., and this classification system (Riddell system) has been widely adopted [27]. Characteristic histological features include mucosal dedifferentiation, disordered differentiation, and increased glandular density. A bottom-up proliferation pattern demonstrated by Ki-67 staining—characterized by proliferative zones located in the basal to middle layers of the crypts—is also observed, whereas in sporadic adenoma the proliferation zone is typically distributed from the surface to the middle layers of the glands (top-down pattern) [28]. In addition, p53 overexpression is frequently detected, even at the low-grade dysplasia stage [29]. These findings may help distinguish UCAN from sporadic CRC or adenoma (Fig. 6). A comprehensive assessment of these macroscopic and histological features is essential for the diagnosis of UCAN. It has long been recognized that the detection of dysplasia in biopsy specimens indicates either synchronous carcinoma elsewhere in the colon or a high risk of rapid malignant transformation [30]. Therefore, early detection of dysplasia as a marker of malignant potential is critical for the timely diagnosis of CRC in patients with UC.

### Prognosis

**Fig. 7:**
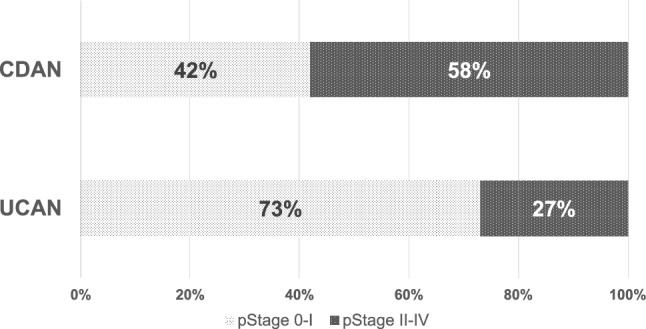
Cancer stages of UC and CD in surveillance cases. The proportion of early-stage cancers (stage 0 or I) was high among patients with UC detected during surveillance, whereas more advanced-stage cancers (stage II, III, or IV) were frequently observed in CD, even during surveillance. UC, ulcerative colitis; CD, Crohn’s disease

Based on its more aggressive histological features, the prognosis of UCAN is worse than that of sporadic CRC, particularly in patients with stage III disease (5-year overall survival [OS]: 43.3% vs. 57.4%, p = 0.032) [23]. A recent JSCCR database study reported a relatively higher 5-year OS rate of 68% among patients with stage III UCAN [9] (Table 1). More intensive surveillance colonoscopy at shorter intervals and the use of biological agents have been associated with a lower incidence of advanced-stage disease (Fig. 7) [9, 31]. In addition to surgical treatment, early detection of UCAN may allow the selection of endoscopic resection, as demonstrated by a recent large-scale Japanese study involving a substantial number of UCAN cases [32]. These findings suggest that appropriate surveillance strategies and optimal use of biological agents are crucial for improving patient outcomes.

### CD-associated neoplasia (CDAN)

#### Epidemiology

Unlike UC, CD presents with diverse manifestations throughout the gastrointestinal tract, including small bowel and perianal lesions; however, carcinogenesis during the long-term disease course is also a major concern. Jess et al. reported that the standardized incidence ratio (SIR) for CRC in CD was 1.9 (95% CI, 1.4–2.5) and 27.1 (95% CI, 14.9–49.2) for small bowel cancer, both significantly higher than that in the general population [2]. Similarly, Canavan et al. reported that the SIR for CRC was 2.5 (95% CI, 1.3–4.7) and 31.2 (95% CI, 15.9–60.9) for small bowel cancer, with cumulative CRC incidences of 2.9% at 10 years, 5.6% at 20 years, and 8.3% at 30 years in patients with CD, with particularly elevated risk among those with colonic involvement [3]. An umbrella review that analyzed 24 studies published between 2005 and 2021 demonstrated that the overall risk of gastrointestinal neoplasia was significantly higher in CD than that in the general population (RR, 1.56; 95% CI 1.10–2.23) [33]. Site-specific analyses revealed markedly elevated risks in the small intestine (RR 11.9; 95% CI, 8.07–17.7), colon (RR 2.30; 95% CI, 1.73–3.06), rectum (RR 1.85; 95% CI, 1.58–2.17), and anus (RR 4.52; 95% CI, 1.19–17.1). Notably, although the RR of small bowel cancer was particularly high, its proportion among all gastrointestinal cancers in CD remained low at approximately 2%.

Regarding predilection sites, regional and ethnic differences have been reported. In Western countries, CD-associated cancers are more frequently observed in the right-sided colon, whereas in Japan, rectal and anal canal cancers, including fistula-associated carcinomas, are more common [34]. A meta-analysis in Asia, including Japan, showed that 84.1% of CD-associated CRC cases were located in the left-sided colon, whereas in Western countries, 63.1% of the cases were located in the left-sided colon [35]. Similarly, a Japanese multicenter study demonstrated that 83% of lesions were located in the left-sided colon, including 48% that involved the anal region (Fig. 5B) (Table 1). Consequently, in Japan, annual surveillance of the anorectal canal is recommended for patients with CD with perianal disease and disease duration of ≥ 10 years. In particular, symptoms, such as increased mucous discharge, bleeding, or worsening pain in the anal region, should raise a suspicion of malignancy and prompt further evaluation.

### Risk factors

Risk factors for CDAN include disease duration, young age at onset, family history of CRC, extensive colonic involvement, and strictures. Long-standing CD has been reported as a risk factor for CRC in CD [3]. Similarly, patients with early-onset CD (before 30 years of age) have been shown to carry a significantly increased risk of developing CD-associated CRC (SIR, 8.2; 95% CI, 1.8–14.6) [36]. In patients with CD having a family history of CRC, the risk of developing CRC is higher compared with those without such a history (RR, 3.7; 95% CI, 1.4–9.4) [15]. Additionally, colonic strictures have been reported to increase the risk of CRC in patients with CD (odds ratio, 8.03; 95% CI, 3.50–18.45) [16]. Regarding anorectal cancer, a Danish nationwide cohort study demonstrated that patients with CD and anorectal fistula have a significantly increased risk of anorectal cancer compared with the general population (hazard ratio, 2.85; 95% CI, 1.80–4.53) [37]. Moreover, chronic perianal disease—particularly long-standing fistulas persisting for more than 10 years—has been recognized as conferring a small but increased risk of perianal and anorectal malignancies, as outlined in a systematic review and expert consensus on perianal fistulizing CD [38]. These findings indicate that perianal disease represents a key site-specific risk factor for CD-associated anorectal cancer and supports the need for careful long-term surveillance.

### Clinicopathological features

**Fig. 8:**
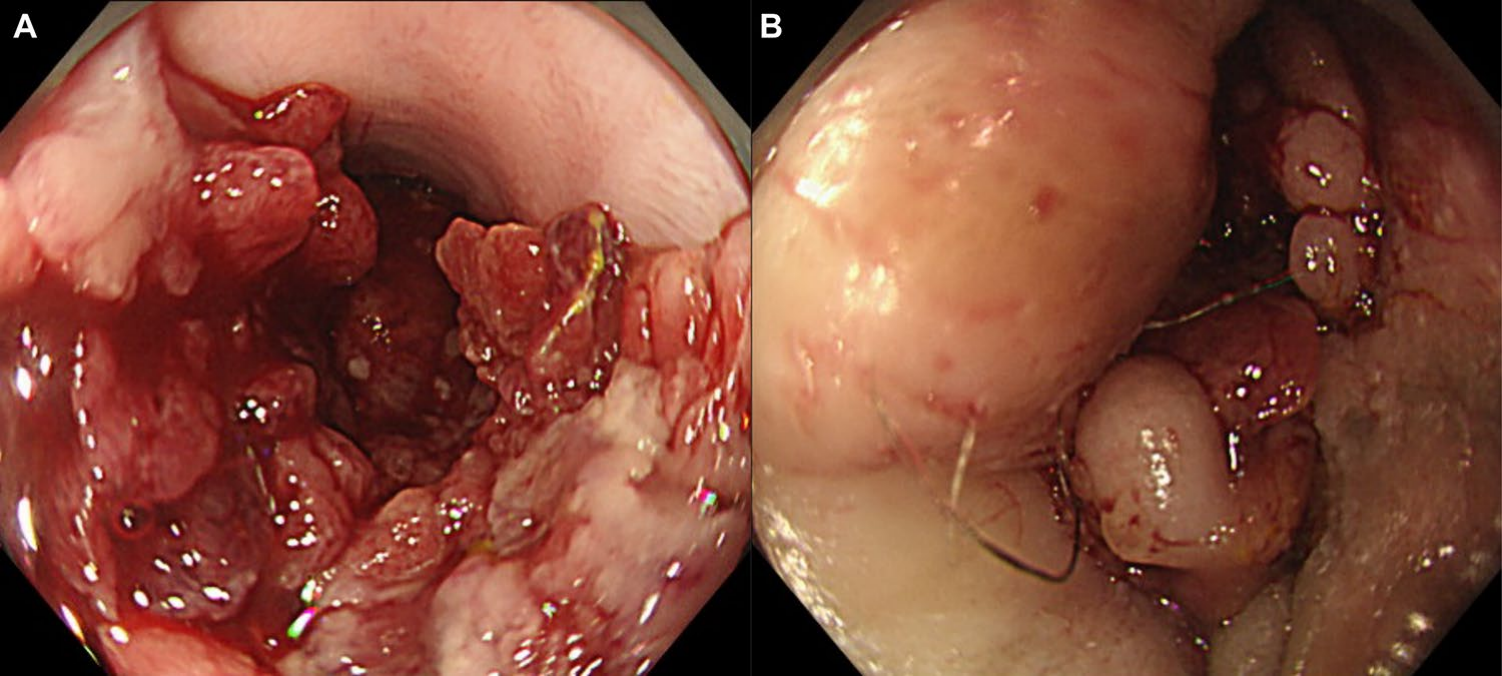
Endoscopic findings of a representative case of CDAN. **A** A protruded villous tumor located in the anorectal region of a patient with CD. CDAN may occasionally exhibit a villous surface pattern. **B** The tumor extends toward the anal side and protrudes outside the anus. Careful inspection of the anorectal region is essential for the early detection of CDAN. CD, Crohn’s disease; CDAN, Crohn’s disease-associated neoplasia

CDAN (including CRC, fistula-associated carcinoma of the anal canal, and small intestinal cancer) often presents with atypical macroscopic features owing to the coexistence of non-neoplastic strictures, fistulas, or inflammatory changes caused by CD. In particular, lesions arising in the rectum and anal canal are frequently accompanied by strictures, and their macroscopic classification is not always consistent (Fig. 8). Another characteristic feature of CDAN is the relatively high incidence of adenocarcinoma, which is thought to arise from fistulas (fistula-associated carcinoma). Histologically, these cancers often exhibit heterogeneous patterns, with a higher frequency of poorly differentiated adenocarcinoma, signet-ring cell carcinoma, and mucinous carcinoma (Fig. 4B) (Table 1).

Pelvic contrast-enhanced computed tomography (CT) and magnetic resonance imaging are useful for assessing advanced fistula-associated cancer, but are not always reliable for detecting early-stage disease. Similarly, PET/CT has limited utility, particularly for mucinous carcinomas, which are relatively common in fistula-associated cancers. Regular and repeated colonoscopic biopsies of indurated areas, secondary fistula openings, fistula tracts, and strictures are essential. In cases in which strictures or pain preclude adequate endoscopic evaluation, examination of the anorectal canal under regional or general anesthesia should be considered. For fistula-associated lesions, seton drainage combined with periodic review and tissue sampling is recommended. Therefore, an early histopathological diagnosis is critical.

### Prognosis

The JSCCR database study reported a significantly worse 5-year OS rate for CDAN than for UCAN, particularly in advanced stages (stage II, 76% vs. 89%, p = 0.01; stage III, 18% vs. 68%, p = 0.0009; and stage IV, 0% vs. 13%, p = 0.04). Moreover, a higher proportion of advanced-stage CDAN cases have been diagnosed even under surveillance than UCAN cases [9] (Fig. 7) (Table 1). Furthermore, the survival outcome of CDAN was significantly poorer than that of sporadic CRC (5-year OS: 54.0% vs. 71.2%, p < 0.001), with a higher local recurrence rate (5-year RFS: 57.9% vs. 71.8%, p = 0.007). As for site-specific prognosis, anorectal cancer is associated with a poorer outcome, with a 5-year OS of 51.6% and a 5-year RFS of 53.0%. Notably, the rate of local recurrence is significantly higher than that of sporadic anorectal cancer (22.3% vs. 5.1%, p < 0.001) [39]. CD-associated small bowel adenocarcinoma also carries a poor prognosis. A systematic review and meta-analysis reported a 5-year OS of approximately 29% (95% CI 18–41%) for CD-associated small bowel adenocarcinoma patients, compared with around 33% for de novo small bowel adenocarcinoma. [40]. Regarding the underlying disease behavior of CD, patients with a penetrating disease phenotype have significantly worse outcomes [41]. Younger age at cancer onset has also been reported as a risk factor for poor prognosis [42]. Similar to UC, early detection through appropriate surveillance and timely therapeutic intervention are essential for improving outcomes in patients with CDAN.

## Conclusion

This review summarizes the epidemiological and clinicopathological features of IBD-associated intestinal neoplasia in patients with UC and CD. These neoplasms exhibit epidemiological and pathological characteristics that differentiate them from those of sporadic CRCs. With the increasing prevalence of IBD and growing clinical importance of neoplastic complications, effective surveillance and early detection remain major challenges. Although the mechanisms underlying carcinogenesis have not yet been fully elucidated, further studies are essential to refine risk stratification, improve diagnostic strategies, and guide timely intervention. A deeper understanding of these processes will ultimately enhance early detection and patient outcomes, thereby shaping future strategies for managing IBD-associated intestinal neoplasia.

## Data Availability

The data underlying this article will be shared after all the analyses are completed and upon reasonable request to the corresponding author.

## References

[1] Ekbom A, Helmick C, Zack M et al (1990) Ulcerative colitis and colorectal cancer. A population-based study. N Engl J Med 323:1228–1233. 10.1056/NEJM199011013231802 2215606 10.1056/NEJM199011013231802

[2] Jess T, Gamborg M, Matzen P et al (2005) Increased risk of intestinal cancer in Crohn’s disease: a meta-analysis of population-based cohort studies. Am J Gastroenterol 100:2724–2729. 10.1111/j.1572-0241.2005.00287.x 16393226 10.1111/j.1572-0241.2005.00287.x

[3] Canavan C, Abrams KR, Mayberry J (2006) Meta-analysis: colorectal and small bowel cancer risk in patients with Crohn’s disease. Aliment Pharmacol Ther 23:1097–1104. 10.1111/j.1365-2036.2006.02854.x 16611269 10.1111/j.1365-2036.2006.02854.x

[4] Hisamatsu T, Miyoshi J, Oguri N et al (2025) Inflammation-associated carcinogenesis in inflammatory bowel disease: clinical features and molecular mechanisms. Cells 14:567. 10.3390/cells14080567 40277893 10.3390/cells14080567 PMC12025475

[5] Ullman TA, Itzkowitz SH (2011) Intestinal inflammation and cancer. Gastroenterology 140:1807–1816. 10.1053/j.gastro.2011.01.057 21530747 10.1053/j.gastro.2011.01.057

[6] Bopanna S, Ananthakrishnan AN, Kedia S et al (2017) Risk of colorectal cancer in Asian patients with ulcerative colitis: a systematic review and meta-analysis. Lancet Gastroenterol Hepatol 2:269–276. 10.1016/S2468-1253(17)30004-3 28404156 10.1016/S2468-1253(17)30004-3 PMC5713894

[7] Olén O, Erichsen R, Sachs MC et al (2020) Colorectal cancer in Crohn’s disease: a Scandinavian population-based cohort study. Lancet Gastroenterol Hepatol 5:475–484. 10.1016/S2468-1253(20)30005-4 32066530 10.1016/S2468-1253(20)30005-4

[8] Japanese Society for Cancer of the Colon and Rectum (2025) JSCCR Guidelines 2024 for the Clinical Practice of Inflammatory Bowel Disease-Associated Intestinal Neoplasia. J Anus Rectum Colon (in press). 10.23922/jarc.2025-071 PMC12854282 41623602

[9] Noguchi T, Ishihara S, Uchino M et al (2023) Clinical features and oncological outcomes of intestinal cancers associated with ulcerative colitis and Crohn’s disease. J Gastroenterol 58:14–24. 10.1007/s00535-022-01927-y 36182971 10.1007/s00535-022-01927-y

[10] Crohn BB, Rosenberg H (1925) The sigmoidoscopic picture of chronic ulcerative colitis (non-specific). Am J Med Sci 170:220–227. 10.1097/00000441-192508010-00006

[11] Eaden JA, Abrams KR, Mayberry JF (2001) The risk of colorectal cancer in ulcerative colitis: a meta-analysis. Gut 48:526–535. 10.1136/gut.48.4.526 11247898 10.1136/gut.48.4.526 PMC1728259

[12] Choi CHR, Rutter MD, Askari A et al (2015) Forty-year analysis of colonoscopic surveillance program for neoplasia in ulcerative colitis: an updated overview. Am J Gastroenterol 110:1022–1034. 10.1038/ajg.2015.65 25823771 10.1038/ajg.2015.65 PMC4517513

[13] Kishikawa J, Hata K, Kazama S et al (2018) Results of a 36-year surveillance program for ulcerative colitis-associated neoplasia in the Japanese population. Dig Endosc 30:236–244. 10.1111/den.12955 28836702 10.1111/den.12955

[14] Jess T, Rungoe C, Peyrin-Biroulet L (2012) Risk of colorectal cancer in patients with ulcerative colitis: a meta-analysis of population-based cohort studies. Clin Gastroenterol Hepatol 10:639–645. 10.1016/j.cgh.2012.01.010 22289873 10.1016/j.cgh.2012.01.010

[15] Askling J, Dickman PW, Karlén P et al (2001) Family history as a risk factor for colorectal cancer in inflammatory bowel disease. Gastroenterology 120:1356–1362. 10.1053/gast.2001.24052 11313305 10.1053/gast.2001.24052

[16] Wijnands AM, de Jong ME, Lutgens MWMD et al (2021) Prognostic factors for advanced colorectal neoplasia in inflammatory bowel disease: systematic review and meta-analysis. Gastroenterology 160:1584–1598. 10.1053/j.gastro.2020.12.036 33385426 10.1053/j.gastro.2020.12.036

[17] Maaser C, Sturm A, Vavricka SR et al (2019) ECCO-ESGAR guideline for diagnostic assessment in IBD part 1: initial diagnosis, monitoring of known IBD, detection of complications. J Crohns Colitis 13:144–164. 10.1093/ecco-jcc/jjy113 30137275 10.1093/ecco-jcc/jjy113

[18] Komatsu K, Shinagawa T, Uchino M et al (2025) Colitis-associated colorectal neoplasia in ulcerative colitis with primary sclerosing cholangitis: a nationwide study. Intest Res10.5217/ir.2025.0013341679315

[19] Laine L, Kaltenbach T, Barkun A et al (2015) SCENIC international consensus statement on surveillance and management of dysplasia in inflammatory bowel disease. Gastrointest Endosc 81:489-501.e26. 10.1016/j.gie.2014.12.009 25708752 10.1016/j.gie.2014.12.009

[20] Anzai H, Hata K, Ishihara S et al (2017) Caution against the resect-and-discard strategy for dysplastic polyps in ulcerative colitis. Am J Gastroenterol 112:189–191. 10.1038/ajg.2016.518 28050047 10.1038/ajg.2016.518

[21] Watanabe T, Ajioka Y, Mitsuyama K et al (2016) Comparison of targeted vs random biopsies for surveillance of ulcerative colitis-associated colorectal cancer. Gastroenterology 151:1122–1130. 10.1053/j.gastro.2016.08.002 27523980 10.1053/j.gastro.2016.08.002

[22] Saito A, Yokoyama Y, Uchino M et al (2025) Analysis of clinicopathological features and oncological outcomes of ulcerative colitis-associated colorectal cancer based on macroscopic classification. Int J Colorectal Dis 40:223. 10.1007/s00384-025-05001-w 41152600 10.1007/s00384-025-05001-w PMC12568850

[23] Watanabe T, Konishi T, Kishimoto J et al (2011) Ulcerative colitis-associated colorectal cancer shows a poorer survival than sporadic colorectal cancer: a nationwide Japanese study. Inflamm Bowel Dis 17:802–808. 10.1002/ibd.21365 20848547 10.1002/ibd.21365

[24] Uchino M, Ikeuchi H, Noguchi T et al (2024) Histological differentiation between sporadic and colitis-associated intestinal cancer in a nationwide study: a propensity-score-matched analysis. J Gastroenterol Hepatol 39:893–901. 10.1111/jgh.16496 38273469 10.1111/jgh.16496

[25] Mizuuchi Y, Nagayoshi K, Nakamura M et al (2024) Prognostic impact of tumour location in stage II/III ulcerative colitis-associated colon cancer: subgroup analysis of a nationwide multicentre retrospective study in Japan. Br J Surg 111:znad386. 10.1093/bjs/znad386 38006321 10.1093/bjs/znad386

[26] Shah SC, Itzkowitz SH (2022) Colorectal cancer in inflammatory bowel disease: mechanisms and management. Gastroenterology 162:715-730.e3. 10.1053/j.gastro.2021.10.035 34757143 10.1053/j.gastro.2021.10.035 PMC9003896

[27] Riddell RH, Goldman H, Ransohoff DF et al (1983) Dysplasia in inflammatory bowel disease: standardized classification with provisional clinical applications. Hum Pathol 14:931–968. 10.1016/S0046-8177(83)80175-0 6629368 10.1016/s0046-8177(83)80175-0

[28] Andersen SN, Rognum TO, Bakka A et al (1998) Ki-67: a useful marker for the evaluation of dysplasia in ulcerative colitis. Mol Pathol 51:327–332. 10.1136/mp.51.6.327 10193513 10.1136/mp.51.6.327 PMC395659

[29] Kobayashi K, Tomita H, Shimizu M et al (2017) p53 expression as a diagnostic biomarker in ulcerative colitis-associated cancer. Int J Mol Sci 18:1284. 10.3390/ijms18061284 28621756 10.3390/ijms18061284 PMC5486106

[30] Morson BC, Pang LS (1967) Rectal biopsy as an aid to cancer control in ulcerative colitis. Gut 8:423–434. 10.1136/gut.8.5.423 6057771 10.1136/gut.8.5.423 PMC1552668

[31] Seishima R, Okabayashi K, Ikeuchi H et al (2023) Effect of biologics on the risk of advanced-stage inflammatory bowel disease-associated intestinal cancer: a nationwide study. Am J Gastroenterol 118:1248–1255. 10.14309/ajg.0000000000002149 36622356 10.14309/ajg.0000000000002149

[32] Hirai M, Yanai S, Kunisaki R et al (2023) Effectiveness of endoscopic resection for colorectal neoplasms in ulcerative colitis: a multicenter registration study. Gastrointest Endosc 98:806–812. 10.1016/j.gie.2023.05.058 37263363 10.1016/j.gie.2023.05.058

[33] Piovani D, Hassan C, Repici A et al (2022) Risk of cancer in inflammatory bowel diseases: umbrella review and reanalysis of meta-analyses. Gastroenterology 163:671–684. 10.1053/j.gastro.2022.05.038 35643170 10.1053/j.gastro.2022.05.038

[34] Choi PM, Zelig MP (1994) Similarity of colorectal cancer in Crohn’s disease and ulcerative colitis: implications for carcinogenesis and prevention. Gut 35:950–954. 10.1136/gut.35.7.950 8063223 10.1136/gut.35.7.950 PMC1374843

[35] Uchino M, Ikeuchi H, Hata K et al (2021) Intestinal cancer in patients with Crohn’s disease: a systematic review and meta-analysis. J Gastroenterol Hepatol 36:329–336. 10.1111/jgh.15229 32865278 10.1111/jgh.15229

[36] Lutgens MW, van Oijen MG, van der Heijden GJ et al (2013) Declining risk of colorectal cancer in inflammatory bowel disease: an updated meta-analysis of population-based cohort studies. Inflamm Bowel Dis 19:789–799. 10.1097/0MIB.0b013e31828029c0 23448792 10.1097/MIB.0b013e31828029c0

[37] El-Hussuna A, Lemser CE, Iversen AT et al (2023) Risk of anorectal cancer in patients with Crohn’s disease and perianal fistula: a nationwide Danish cohort study. Colorectal Dis 25:1453–1459. 10.1111/codi.16581 37086006 10.1111/codi.16581

[38] Wong SY, Rowan C, Brockmans ED et al (2025) Perianal fistulizing Crohn’s disease-associated anorectal and fistula cancers: systematic review and expert consensus. Clin Gastroenterol Hepatol 23:927-945.e922. 10.1016/j.cgh.2024.05.029 38871152 10.1016/j.cgh.2024.05.029

[39] Ogino T, Mizushima T, Fujii M et al (2023) Crohn’s disease-associated anorectal cancer has a poor prognosis with high local recurrence: a subanalysis of the nationwide Japanese study. Am J Gastroenterol 118:1626–1637. 10.14309/ajg.0000000000002269 36988310 10.14309/ajg.0000000000002269 PMC10453357

[40] Chin YH, Jain SR, Lee MH et al (2022) Small bowel adenocarcinoma in Crohn’s disease: a systematic review and meta-analysis of the prevalence, manifestation, histopathology, and outcomes. Int J Colorectal Dis 37:239–250. 10.1007/s00384-021-04050-1 34704127 10.1007/s00384-021-04050-1

[41] Yamamoto A, Toiyama Y, Ikeuchi H et al (2023) Oncological outcomes of Crohn’s disease-associated cancers focusing on disease behavior. Ann Gastroenterol Surg 7:615–625. 10.1002/ags3.12653 37416732 10.1002/ags3.12653 PMC10319610

[42] Okita Y, Toiyama Y, Ikeuchi H et al (2024) Possible poor prognosis in younger-onset Crohn’s disease-associated anorectal cancer: a subanalysis of the nationwide Japanese study. Ann Gastroenterol Surg 8:620–630. 10.1002/ags3.12773 38957565 10.1002/ags3.12773 PMC11216786

